# Assessment of the Driving Pollution Factors of Soil Environmental Quality Based on China’s Risk Control Standard: Multiple Bigdata-Based Approaches with Intensive Sampling

**DOI:** 10.3390/ijerph191912459

**Published:** 2022-09-30

**Authors:** Xiahui Wang, Nan Wei, Guohua Ji, Ruiping Liu, Guoxin Huang, Hongzhen Zhang

**Affiliations:** Center for Soil Protection and Landscape Design, Chinese Academy of Environmental Planning, Beijing 100012, China

**Keywords:** soil environmental quality, cultivated land, heavy metals, driving factor, environment standard

## Abstract

Identifying the driving factors of soil environmental quality is critical in raising countermeasures for managing the soil environment efficiently and precisely. In 2018, China issued risk control standards to divide soil environmental quality into three classes to meet the demands of environment management. However, there is a lack of knowledge of this new standard. An intensive field-sampling research (2598 top-soil samples were analyzed) was conducted in the agricultural land of Hubei province, central China, to find the driving factors of pollutants based on this new standard. According to the standard, the proportion of classes 1, 2, and 3 in the overall quality grade was 57.3%, 41.7%, and 1%, respectively. The standardized index showed that the pollution levels of cadmium, arsenic, lead, and chromium were higher than that of mercury. The first component of the principal component analysis explained 56.4% of the total variance, and the loading of cadmium, arsenic and lead were −53.5%, −52.1%, and −51.2%, respectively. The general linear modeling found that cadmium and arsenic showed positive and significant effects (*p* < 0.001) on the grading results of soil environmental quality. Based on the random forest algorithm, cadmium showed the greatest importance on soil environmental quality (increase in mean squared error = 32.5%). Overall, this study revealed that cadmium, arsenic, and lead were driving pollutants affecting soil environment quality grade. The large data size increased the reliability and robustness of the study’s conclusions, and it provided reference methods for future studies investigating China’s new standard for assessing soil environmental quality.

## 1. Introduction

Soil environmental quality assessment is an essential part of total environmental quality assessment because environmental quality includes air, water, and soil quality [1]. Because of the heterogeneity and chronicity of soil pollution, soil assessment quality differs from air and water assessment quality [2]. The concept of soil quality started in 1971 [3], then combined with policymaker demand [4,5], assessment of environmental risk [6], and soil remediation [7]. Developing assessment methods and grading the soil environmental quality are important topics in the field of soil research [8,9,10,11].

The method focuses on the physical, chemical, and biological parameters of soil [9,12,13,14,15]. The minimum data set includes 6–8 parameters to increase the feasibility and economic techniques [16,17,18,19,20]. The parameters will be integrated and classified into different grades to meet the demand for environmental management and land functions. The driving factors of soil quality grading may differ depending on the assessment methods used.

Heavy metals can accumulate in the surface soil through various pathways, including bedrock mineralization, atmospheric sedimentation, and human activities. The accumulation process is chronic, invisible, irreversible, and temporally lagged [21,22,23] because of the properties of the soil. Since human industrial and agricultural development activities, the accumulation of heavy metals in soils has drawn attention to serious concerns on human health [24,25,26,27,28,29,30,31] and ecosystems [32]. Several index methods, such as Muller’s geoaccumulation index [33], Nemerow’s index [34], and Hakanson’s ecological risk index [35], have been developed to assess the quality or risk of soil caused by heavy metals. In recent decades, researchers kept improving these assessment methods to meet the changing demands for soil environmental protection. Several bigdata-based approaches were used to investigate the factors of soil quality, such as minimum data set, principal component analysis (PCA), general linear model (GLM), and random forest (RF). Compared with PCA and GLM, the RF algorithm [36] is a new bigdata-based approach developed from the classification and regression tree (CART) model [37,38]. RF is less sensitive to model overfitting than CART. Thus, it contains both a single regression tree as well as multiple regression trees. RF has a rapid calculation time and does not require complex data processing [39,40]. Furthermore, RF can analyze the importance of variables, making the results of driving factors more interpretable.

China started to handle its soil environmental issues caused by rapid economic and industrial development at the end of the 20th century, thus, published a series of regulations and standards related to the soil environment. In 2016, China published a soil pollution prevention and control action plan (hereinafter referred to as the action plan, State Council of China, 2016) to control its soil pollution and improve the quality of the soil environment on a long-term scale (2016–2050). Following the implementation of the action plan, a series of new regulations, standards, and guidelines related to soil environment and pollutants were issued or updated. The Ministry of Ecology and Environment (hereinafter referred to as MEE China) issued a new assessment and grading standard for agricultural soil environmental quality in mid-2018 [41], which mainly focuses on soil pollutants, especially heavy metals.

At present, several studies have investigated or reviewed the principal heavy metals in the soil at the national level or regional level based on different guideline values [42,43,44,45,46,47]. However, there is a lack of knowledge about China’s newly issued standard, especially for intensive sampling surveys, which could improve the reliability of bigdata analysis. Therefore, we conducted an intensive field survey on cultivated land located in a mining area of Hubei province, which is characterized by mining activities and industrial activities [45]. Based on China’s newly issued risk control standard, we analyzed the driving pollutant factor of soil environmental quality using multiple bigdata-based approaches.

## 2. Materials and Methods

### 2.1. Assessment and Grading

#### 2.1.1. Standard of Soil Pollutants Assessment

The assessment and grading methods follow the soil environmental quality risk control standard for agricultural land soil contamination (hereinafter referred to as risk control standard [41]). Table 1 and Table 2 show risk-screening and risk-intervention values for each pollutant factor according to the soil pH value and utility functions. Only five major soil pollutants, including cadmium (Cd), mercury (Hg), arsenic (As), lead (Pb), and chromium (Cr), were listed for the risk-screening and risk-intervention values. Therefore, in this study, we assessed five major soil pollutants and one soil property parameter (pH value) according to the risk control standard.

#### 2.1.2. Grading of Soil Environmental Quality

According to the action plan and risk control standard, the grades of soil environmental quality of agricultural land were classified into three classes: conservation priority (class 1, considered as low risk), safety usage (class 2, considered as controllable risk), and strict control (class 3, considered as high risk). Class 1 indicates that the soil environmental risk is low enough to be considered safe for agricultural products and human health; class 2 indicates that soil environmental risk exists and can jeopardize the safety of agricultural products, and class 3 indicates that the soil environment risk is remarkably high and strict countermeasures should be implemented.

As shown in Table 3, in the case of grading soil quality for a single pollutant factor, if the content is lower than the risk-screening value, the grade is class 1. If the pollutant content is between the risk-screening and risk-intervention value, the grade will be class 2. If the pollutant content exceeds the risk-intervention value, the grade will be determined as class 3. The highest class (highest risk) of single pollutant factors is used to determine the overall grade of the soil sample.

### 2.2. Field Sampling and Chemical Analysis

#### 2.2.1. Field Sampling

The investigated area is in a county of Hubei province in central China. The area spans 76 km from west to east and 71 km from south to north, with a total area of more than 600 km^2^. The climate is subtropical monsoonal, with annual precipitation and temperature of 1389 mm and 16.8 °C, respectively. Fluvo-aquic soil sand Anthrosol is the prevailing soil type formed from river and lake sediments, quaternary clay, and quartz sandstone. In this area, the land is mainly used for planting rice, vegetables, and other economic crops. According to the available information from residents and other historical materials, the investigated area has a large number of mineral reserves of copper and gold.

**Figure 1:**
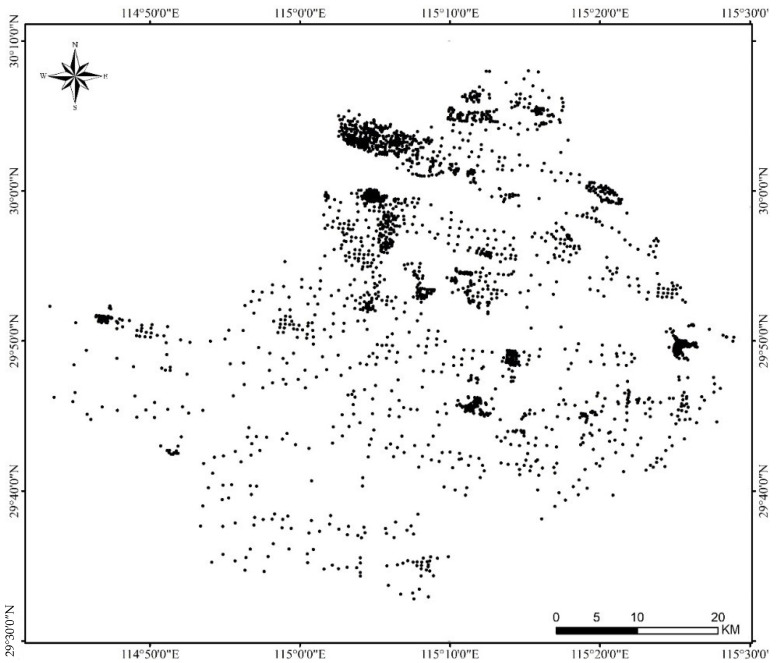
Study area and location of the sampling sites.

From September 2018 to November 2018, surface soil samples (0–20 cm) were collected using a random sampling scheme (Figure 1). The geographic coordinates of each sampling site were recorded using a handheld global positioning system. At each sampling site, five top-soil samples (500 g each, depth of 0–20 cm) were taken, and five top-soil samples were mixed as one field sample. In total, 2598 field samples (sites) were taken in cultivated land, including 1187 paddy field soil samples and 1411 other soil samples. Approximately 1 kg of fresh soils were collected, packed in plastic bags, and transported back to the laboratory. All collected soil samples were air-dried at room temperature, ground, and sieved.

#### 2.2.2. Chemical Analysis

The chemical analysis was conducted at Chengdu Supervision and Inspection Center of Mineral Resources, Ministry of Natural Resources. The chemical analysis of soil samples was conducted according to the methods suggested by the MEE *risk control standard* [41]. After air-drying, soil samples were ground for subsequent chemical analysis. For determination of pH value, soil samples were sieved through nylon mesh with 2 mm size, then 10 g of sieved sample was mixed with 25 mL water and measured using pH electrode. For the determination of heavy metals, soil samples were sieved through a nylon mesh of 0.149 mm size, then microwave-assisted for aqua regia digestion. The total contents of Cd, Pb, and Cr were measured using inductively coupled plasma mass spectrometry (ICP-MS), while the total contents for Hg and As were measured using atomic fluorescence spectroscopy.

#### 2.2.3. Quality Control

The MEE guidelines for environmental analytical methods (HJ 168-2010, MEE, 2010 [48]) were used to assess the limit of quantification (LOQ), the limit of detection (LOD), spiking recovery rate, and the degree of precision of chemical analysis methods used in this study. In this study, two blank samples were tested as negative controls, with approximately every 100 soil samples for 26 negative controls.

### 2.3. Data Analysis

#### 2.3.1. Intervention Value-Based Pollutant Index of Pollutant Factors

Because of the large difference in the standard value (Table 1 and Table 2) for different pollutant factors, the index of pollutant factors was calculated by normalizing with risk-intervention values (Table 2). Equation (1) describes the equation for calculating the index.

(1) $$P_{i}=\frac{C_{i}}{S_{i}}$$

where *P_i_* is the index of a certain pollutant; *C_i_* is the measured content of a certain pollutant, and *S_i_* is the risk-intervention value of a certain pollutant. 

#### 2.3.2. Statistical Analysis

To study the driving pollutant factors of soil environmental quality grades, PCA was applied to the contents of Cd, Hg, As, Pb, and Cr in our soil samples. PCA was used in R 3.6.3 [49] by function “prcomp” with parameter “scale = TRUE.”

GLM was used to analyze the relationship between overall soil environmental quality grade and soil pollutants. The pH value and log10 scaled contents of Cd, Hg, As, Pb, and Cr were set as independent variables, while the soil environmental quality grade (class 1, 2, and 3) was set as the dependent variable.

The RF algorithm has three tuning parameters: the number of input variables randomly selected as candidates at each split (mtry), the number of trees (ntrees), and minimum node size (nodesize). The package’s default ntrees value is 500. For node size, the standard for regression analysis is five for each terminal node. The lowest out-of-bag error estimate was used to determine the optimal value of mtry. The RF algorithm was performed in R using the “randomForest” package [50], with the pH value and contents of Cd, Hg, As, Pb, and Cr set as independent variables, and the soil environmental quality grade (class 1, 2, and 3) set as the dependent variable.

All the data analysis and visualization studies were performed on the platform of RStudio 2021.09.1 [51] and R 4.0.4 [49].

## 3. Results

### 3.1. Pollutant Content

**Figure 2:**
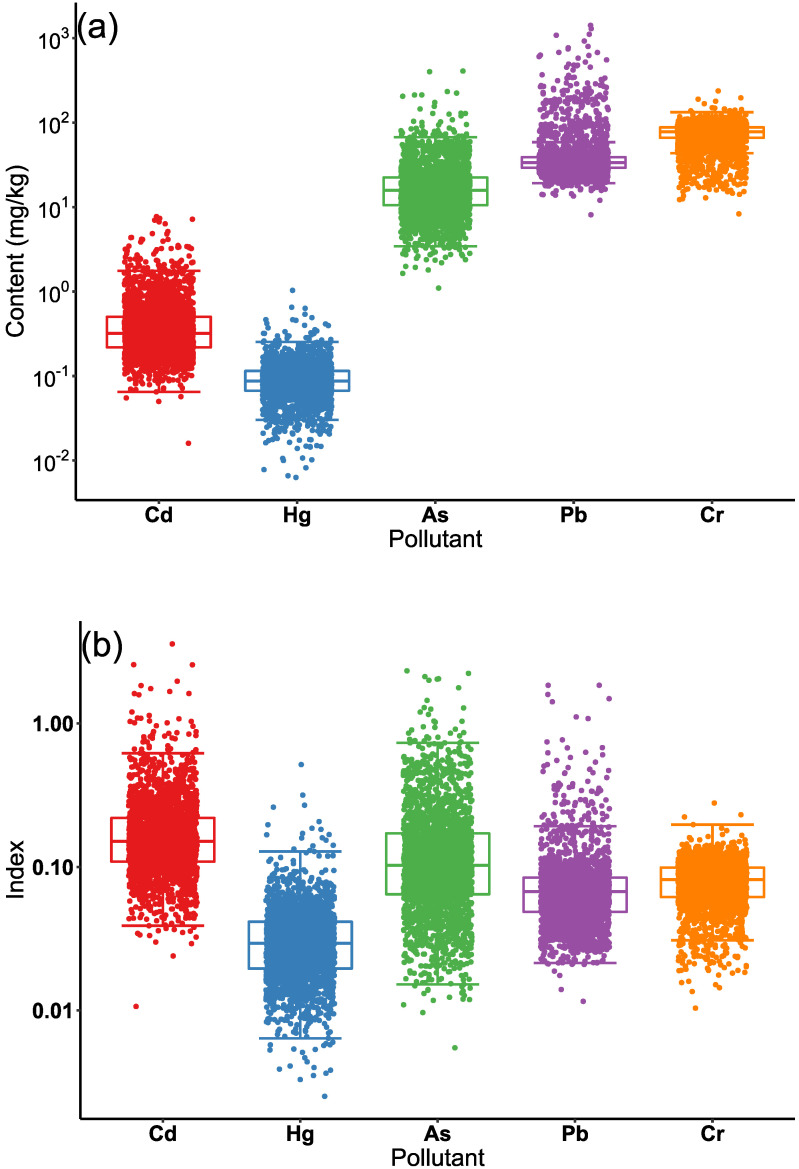
The boxplot of each pollutant content (**a**) and index (**b**) in soil samples. Each dot represents one soil sample. The data was shown in log10 scale.

**Figure 3:**
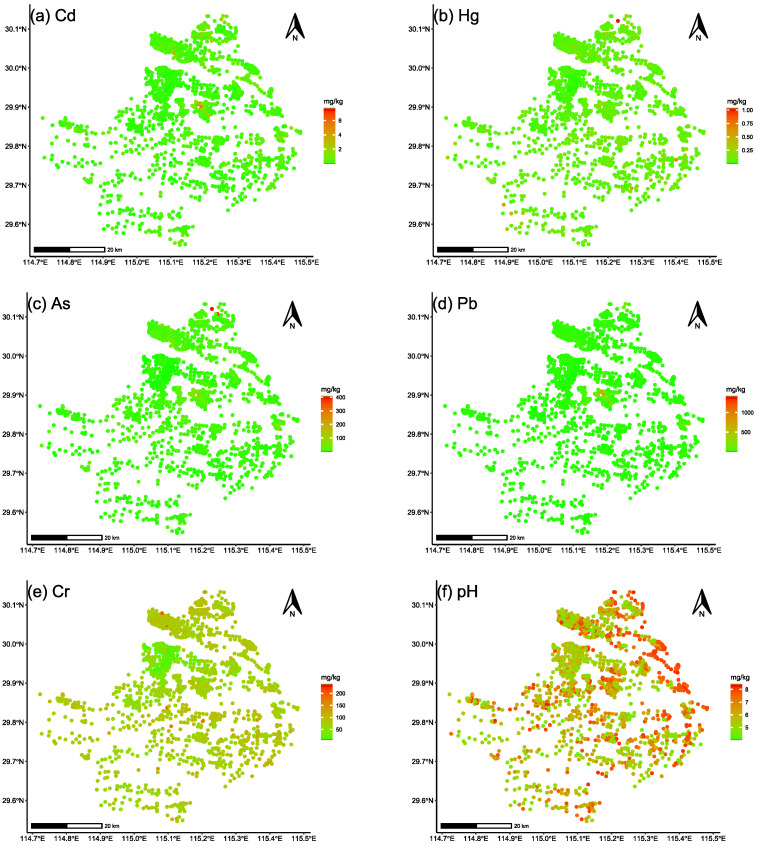
The distribution map of heavy metals and pH in the study area.

In Figure 2, the content and index of each pollutant are shown in a boxplot with log10 transformation. The distribution map of heavy metals and pH are shown in Figure 3, and the descriptive statistics for each pollutant are shown in Table 4. The content and variability of As, Cd, Pb, and Cr were higher than those of Hg (Figure 2a, Table 4). The index and variability of As, Cd, Pb, and Cr were higher than those of Hg (Figure 2b, Table 4), indicating that the As, Cd, Pb, and Cr pollutant levels were high according to the MEE risk-intervention value [41] (Table 2). 

### 3.2. Soil Environmental Quality Grading

**Figure 4:**
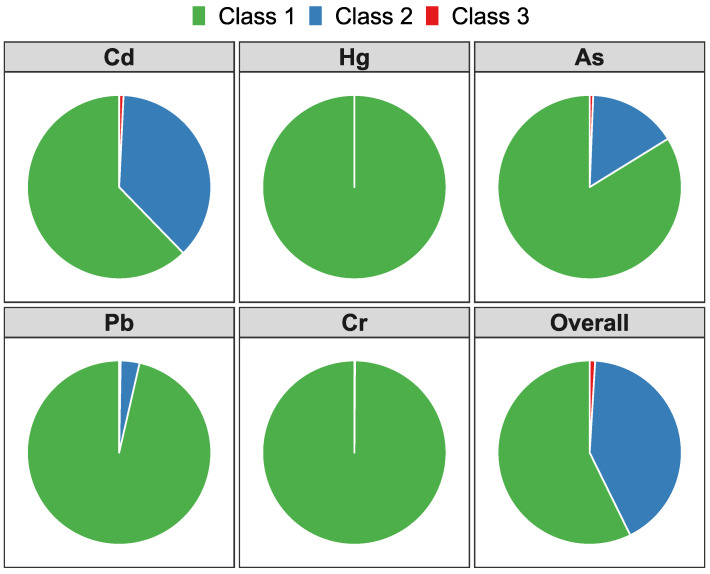
The proportion of each soil environment quality class by single pollutant factor and multiple factors (overall grade).

Figure 4 shows the proportions of each soil environmental quality grade by single pollutant factor and multiple factors (overall grade). According to the MEE *risk control standard*, the proportion of classes 1, 2, and 3 in the overall quality grade was 57.3%, 41.7%, and 1%, respectively. In the case of single pollutant factors, Hg and Cr graded 100% and 99.9% of soil samples as class 1, respectively. Cd and As showed a lower proportion of class 1 (62.3% and 83.8%) than Hg, Pb, and Cr. Cd, As, and Pb graded 0.8%, 0.6%, and 0.3% soil samples as class 3, respectively. Hg or Cr did not grade any soil sample as class 3.

### 3.3. PCA, GLM, and RF Analysis

**Figure 5:**
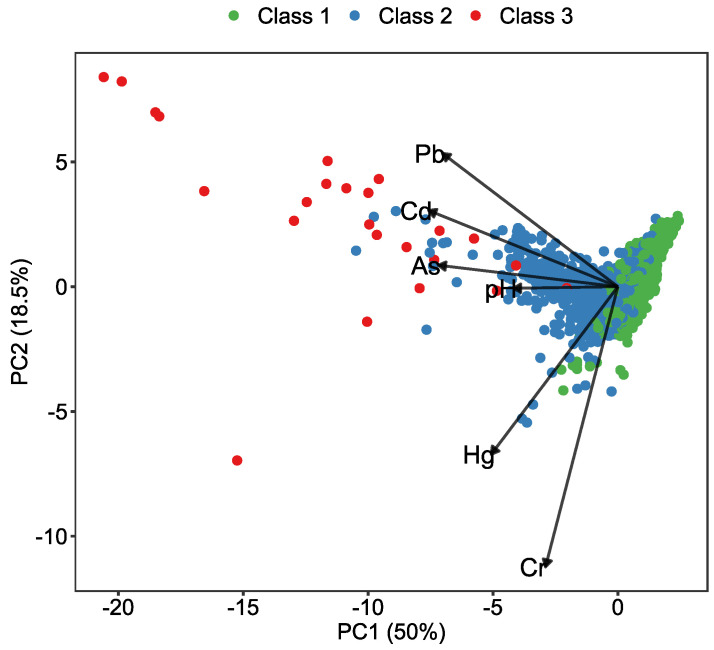
The projection of pollutant contents on the 1st component (PC1, 56.4% variance) and the 2nd component (PC2, 22.2% variance). The arrow stands for the vector of each pollutant factor. Each dot represents one soil sample.

According to the PCA results, the Cd, Hg, As, Pb, and Cr contents could be projected to two components, which accounted for 78.6% of the total variance in the data (Table 5). The soil environmental quality grade was roughly separated by the axis of the 1st component (PC1) (Figure 5), which explained 56.4% of the total variance, the loadings of Cd, As, and Pb were −53.5%, −52.1%, and −51.2%, respectively. According to the PCA results, Cd, As, and Pb were associated with high loadings in PC1 and were the main factors of soil environmental quality grade.

According to the GLM regression (Table 6), the contents of Cd, Hg, As, Pb, Cr, and pH showed a significant effect (*p* < 0.001) on the grading results of soil environmental quality. In our field samples, the contents of Cd and As showed positive effects on soil environmental quality, indicating the contents of these two heavy metals were positively correlated to soil environment quality grade, while the content of Hg, Pb, Cr, and pH value showed negative effects, indicating these factors were negatively correlated to soil environment quality grade.

**Figure 6:**
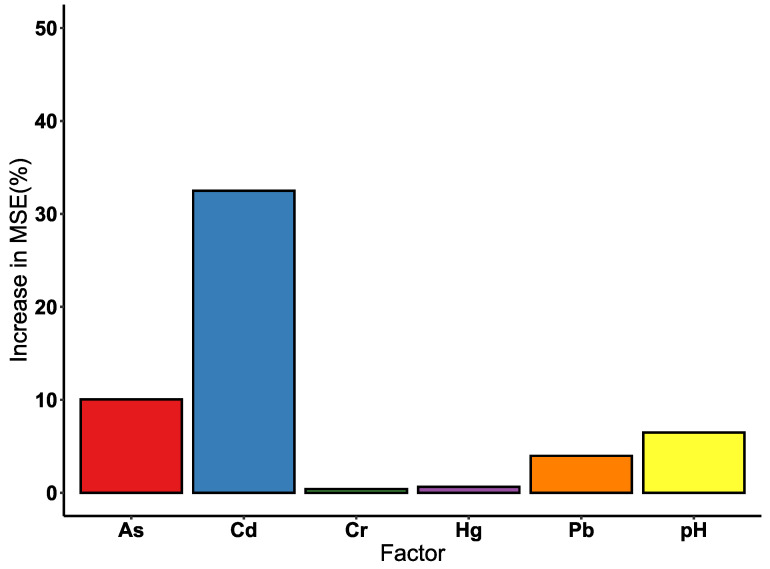
The increase in mean squared error for soil environment quality of each factor in soil samples.

The RF algorithm showed an increase in mean squared error and node purity of Cd, Hg, As, Pb, Cr, and pH (Figure 6 and Table 7), indicating the importance of these soil factors on soil quality. Among the factors investigated in this study, Cd showed the greatest importance on soil environmental quality (increase in mean squared error = 32.5%, increase in node purity = 341.765). Compared with other factors, As showed relatively high importance on soil environmental quality (increase in mean squared error = 10.0%, increase in node purity = 146.863). Based on RF algorithm results, pH, Pb, Hg, and Cr did not show significant importance on soil environmental quality.

## 4. Discussion

### 4.1. Pollutants in the Survey Area

In our study area, the soil environmental quality of most samples was graded as conservation priority class and safety usage class (Figure 4), indicating that the pollution risk in the surveyed area was acceptable and controllable based on the MEE *risk control standard* [41]. The intervention value-based pollutant index (Figure 2b) implied that the pollution risk of Cd, As, Pb and Cr were higher than that of Hg, which was generally consistent with PCA results. According to the historical records of our study area, there have been rich minerals and active mining activities of copper and gold for hundreds of years. Thus, the associated minerals could be one of the major causes of high Cd, As, and Pb levels (Figure 3a,c,d). Because of the large difference in the criteria values of these pollutants, the pollutant content (Figure 2a) concealed the pollution level of Cd and exaggerated the pollution level of Cr to some extent. Therefore, compared with the pollutant content, the intervention value-based pollutant index could more accurately identify severe pollutants because the index was normalized by standard values.

Only the total heavy metal contents were analyzed in this study because of the risk control standard and our large sample size. However, the valence and forms of heavy metals (e.g., Cr-VI and Cr-III) have different environmental risks for agricultural land and products. Only the total contents of some major heavy metals were incorporated into the risk control standard as a tradeoff between cost and technique because China completed soil quality-grading work for agricultural land on a national scale at the county level in the coming years and because of a large number of soil samples (million class). With advancements in economy and technology, a more precise indicator such as various valence and forms of heavy metals should be incorporated into an updated version of this standard in the future to better protect and manage the soil environment.

### 4.2. The Driving Pollutant Factors

In this study, the PCA results (Table 5) revealed the possible homology of Cs, As, and Pb, implying that the pollution source of these three pollutants may also be homologous (Figure 3a,c,d). Several recent studies have used PCA to investigate the sources of heavy metals in soil. Jin et al. [52] used PCA to identify the latent constructs that controlled heavy metal variability and reflected potential sources at children’s playgrounds in Beijing, China. Yang et al. [53] used PCA to determine that four mine sources contributed 89.8% of heavy metal accumulations in Hubei, China. Because our study area is also rich in minerals, the sources of these three pollutants are mineral resources and mining activity.

Furthermore, the mapping results associated with soil environmental quality grades revealed that Cd, As, and Pb were the main driving factors of soil environmental quality. The GLM results showed that the contents of Cd and As were positively related to the grade class, implying that treating Cd and As would be beneficial for improving soil environmental quality. Meanwhile, the RF algorithm results also showed a similar interpretation of GLM results, indicating that Cd and As were the driving factors of soil environmental quality. However, the GLM and RF results did not show any obvious effect on soil environmental quality from Pb, which differs from PCA results. One possible reason was that the soil quality of Pb was much better than that of Cd and As (Figure 4), and the dependent variable (grade class, discrete variable in reality) of GLM and RF was set as a continuous variable, causing regression to be slightly distorted.

Several studies also investigated the driving factors of heavy metals in soil, both at the national level and regional levels. In the case of national-level studies, Chen et al. [44] reviewed 779 topsoil studies published from 2009 to 2020 and conducted a fuzzy eco-health risk model, identifying the risks from Pb, Cd, As, and Hg as the priority control metals at the national level in China. Moreover, Cd and Hg were the principal pollution factors in the region of Hubei province, which was partly consistent with our study since our study area was also located in Hubei province. Tóth et al. [47] analyzed soil-heavy metals from 22,000 locations in the European Union (LUCAS topsoil database) based on the Finnish legislation for contaminated soil [54]. Approximately 6.24% of the agricultural land needs local assessment and eventual remedial action. The proportion of samples that surpassed the threshold value for Cd and Cr were 5.5% and 4.4%, respectively. Due to the fact that the risk-intervention values of MEE China (Table 2) are much higher than their counterparts of MEF Finland, the proportion of Class 3 (approximately 1%) in our samples was much lower than the proportion of samples that needs assessment or remedial action (approximately 6.24%) in Toth’s samples. Barsova et al. [42] reviewed surveys compiled from 2008 to 2012 in the Russian Federation, reporting that the average contents of mobile species of heavy metals (Cu, Zn, Cd, Pb, Ni, Cr, Hg) were several times lower than the allowed maximum permissible concentrations [55], and reported a decreasing trend both in the country scale and federal districts scale. The soil environmental quality of the Russian Federation was better than that of the European Union and China. A possible reason was that the population density and human activity were relatively low in the Russian Federation. In the case of regional-level studies conducted in a mining area, Bech et al. [43] investigated a copper mine area in Northern Peru and confirmed As and Cu were the main pollution factors in this area. Mireles et al. [46] collected soil samples from a mining area in the state of Zacatecas, Mexico. They reported that when the content of As, Ba (barium), Cr, Fe (ferrum), Mn (Manganese), and Zn (zinc) were compared with the guidelines of the US EPA [56] the urban soils turned out to be heavily polluted, while the content of Ba and Cr were lower than residential Mexico Guideline values [57] since the differences in the elemental concentrations between guidelines of US EPA and guidelines of Mexico were large.

### 4.3. Environmental Management Suggestions Based on Soil Environmental Quality Assessment

Jennings [58] analyzed worldwide regulatory guidance values for commonly regulated heavy metals in surface soil: Pb, Cd, As, Ni, Cr, Hg, Cu, and Zn were the most frequently regulated elements. Most countries or regions in North America, the European Union, and Eastern Asia have regulatory values for the total content of Cd, Hg, As, and Pb. Except for China, valent state-based regulatory values of Cr (Cr-VI and Cr-III) were also used in these countries or regions. Moreover, China’s *risk control standard* only incorporated the total contents of Cd, Hg, As, Pb, and Cr into risk-intervention values; hence our study only tested the total contents of these five heavy metals. However, most global soil guidelines or standards did not classify soil into different categories based on regulatory value, as China’s *risk control standard* did. In this study, we propose some environmental management suggestions based on previous research and agricultural land regulations in China [41]. To protect the less-polluted soil on agricultural land where the conservation priority class soil is in the majority, nearby industries such as nonferrous metal smelting, petroleum refining, electroplating, etc., should be strictly restricted. To ensure the safety of agricultural products from potential pollution risks on agricultural land where the safety usage class soil is in the majority, measures such as substitute plantation, crop rotation, intercropping, etc., should be implemented when necessary. Any agricultural cultivation activity is not recommended for agricultural land where the strict control class soil is in the majority, and intervention measures such as the grain for the Green Project should be taken.

Our study determined that Cd, As, and Pb were driving pollutant factors and showed homology to some extent, implying that treating these three driving pollutants in our study area’s soil would effectively improve soil environmental quality. The environmental risk of Cd, As, and Pb in the soil has significant toxicity to plants [59,60,61] in aspects of ecology and physiology. Therefore, we propose environmental management techniques such as controlling emission sources (probably mining activity) and conducting soil remediation of these three driving pollutants. Remediation technologies such as agronomical measures and phytoremediation have been developed to treat agricultural soil contaminated by heavy metals. Proper agronomical measures such as adjustment of cropping pattern and fertilization could alleviate the heavy metal pollution in agricultural land; planting hyperaccumulators such as *Eremochloa ciliaris*, *Solanum nigrum* L., and *Sedum alfredii* could remove As, Cd, and Pb from the soil.

China’s *risk control standard* for soil environmental quality mainly focused on soil pollutants such as the total content of heavy metals, with no consideration given to valence states of heavy metals and soil fertility indicators such as soil organic matter and total nitrogen. To improve soil environment management, it is necessary to apply more indicators to regional standards, such as soil fertility indicators and different valence states of heavy metals, considering the regional differences in soil characteristics. To further evaluate this standard, more comparative studies between this standard and present major soil quality assessment index methods, such as the Muller geoaccumulation, Nemerow, and Hakanson’s ecological risk indices, should be conducted in the future.

## 5. Conclusions

This study conducted an intensive field research sampling and assessed the soil environmental quality based on China’s newly issued *risk control standard* [41]. The large data size increased the reliability and robustness of the study’s conclusions. The intervention value-based pollutant index showed that Cd, As, Pb, and Cr pollution levels were higher than that of Hg. Meanwhile, PCA revealed that the driving pollutants on soil environmental quality grade were Cd, As, and Pb. GLM and RF agree that Cd and As had significant effects on soil environmental quality. Therefore, treating Cd, As, and Pb pollutants were critical to improving the soil environmental quality in this study area. This study used several bigdata-based analytical methods to find driving pollutants based on China’s newly issued *risk control standards*, which should provide some context for researchers to further study this new standard for soil environmental quality.

## Figures and Tables

**Table 1:** Risk-screening value of soil pollutant factors for agricultural land (MEE China, 2018).

Pollutant Factor	Utility Function	Risk-Screening Value (Total Content, mg·kg^−1^)			
		pH Range			
		Lower than 5.5	5.5 to 6.5	6.5 to 7.5	Higher than 7.5
Cd	Paddy	0.3	0.4	0.6	0.8
	Others	0.3	0.3	0.3	0.6
Hg	Paddy	0.5	0.5	0.6	1.0
	Others	1.3	1.8	2.4	3.4
As	Paddy	30	30	25	20
	Others	40	40	30	25
Pb	Paddy	80	100	140	240
	Others	70	90	120	170
Cr	Paddy	250	250	300	350
	Others	150	150	200	250

**Table 2:** Risk-intervention value of soil pollutant factors for agricultural land (MEE China, 2018).

Pollutant Factor	Risk-Intervention Value (Total Content, mg·kg^−1^)			
	pH Range			
	Lower than 5.5	5.5 to 6.5	6.5 to 7.5	Higher than 7.5
Cd	1.5	2	3	4
Hg	2	2.5	4	6
As	200	150	120	100
Pb	400	500	700	1000
Cr	800	850	1000	3000

**Table 3:** Grading of soil environment quality.

Pollutant Content	Class of Single Factor					Overall Class
	Cd	Hg	As	Pb	Cr	
Lower than risk-screening value	1	1	1	1	1	Determined by the highest class of single pollutant factor
Between risk-screening value and risk-intervention value	2	2	2	2	2	
Higher than risk-intervention value	3	3	3	3	3	

**Table 4:** Descriptive statistics of pollutants and pH in soil samples.

	Pollutant	Mean	Median	Minimum	Maximum	SD	CV	Skewness	Kurtosis
		mg·kg^−1^							
Content	Cd	0.48	0.32	0.02	7.71	0.58	121.06%	5.68	50.59
	Hg	0.10	0.09	0.01	1.03	0.05	55.87%	4.58	53.78
	As	19.89	15.81	1.10	407.65	20.28	101.96%	8.24	122.62
	Pb	49.19	33.61	8.11	1416.33	77.73	158.02%	9.13	114.67
	Cr	75.10	77.71	8.31	236.80	21.43	28.54%	−0.28	5.72
Index	Cd	0.19	0.15	0.01	3.59	0.18	92.62%	7.35	95.79
	Hg	0.03	0.03	0.00	0.52	0.02	69.70%	6.27	93.87
	As	0.15	0.10	0.01	2.33	0.18	115.97%	5.27	48.06
	Pb	0.08	0.07	0.01	1.85	0.10	119.27%	10.56	155.77
	Cr	0.08	0.08	0.01	0.28	0.03	32.95%	0.25	4.54
	pH	6.20	5.89	4.05	8.40	1.12	18.01%	0.40	1.83

**Table 5:** Factor loadings, extraction sums of squared loadings, and the proportion of variance.

	PC1	PC2
Cd	−0.535	0.210
Hg	−0.368	−0.464
As	−0.521	0.058
Pb	−0.512	0.367
Cr	−0.206	−0.775
Standard Deviation	1.679	1.055
Proportion of Variance	56.4%	22.2%
Cumulative Proportion	56.4%	78.6%

**Table 6:** Results of GLM analysis showing the effects of contents of Cd, Hg, As, Pb, Cr, and pH on the grading results of soil environment quality (class 1, 2, 3). The data of heavy metal contents were standardized by log10 transformation.

	Coefficient	Standard Error	t-Value	*p*-Value
Intercept	2.853	0.163	17.483	<0.001
Cd	1.378	0.040	34.261	<0.001
Hg	−0.209	0.042	−4.909	<0.001
As	0.689	0.042	16.498	<0.001
Pb	−0.462	0.048	−9.527	<0.001
Cr	−0.215	0.056	−3.808	<0.001
pH	−0.114	0.007	−15.495	<0.001

**Table 7:** Results of RF algorithm showing the importance of contents of Cd, Hg, As, Pb, Cr, and pH on the grading results of soil environment quality (class 1, 2, 3).

Factors	Increase in Mean Squared Error (%)	Increase in Node Purity
Cd	32.5	341.765
Hg	0.6	28.035
As	10.0	146.863
Pb	4.0	62.480
Cr	0.4	18.004
pH	6.5	79.173

## Data Availability

The datasets used and analyzed during the current study are available from the corresponding author upon reasonable request.
